# PIG’s Speed Estimated with Pressure Transducers and Hall Effect Sensor: An Industrial Application of Sensors to Validate a Testing Laboratory

**DOI:** 10.3390/s17092119

**Published:** 2017-09-15

**Authors:** Gustavo F. Lima, Victor C. G. Freitas, Renan P. Araújo, André L. Maitelli, Andrés O. Salazar

**Affiliations:** 1 Instituto Federal do Rio Grande do Norte, Rua Antônia de Lima Paiva, 155, Nova Esperança, Parnamirim CEP 59143-455, RN, Brazil; victor.carvalho@ifrn.edu.br; 2 Departamento de Engenharia de Computação e Automação, Universidade Federal do Rio Grande do Norte, Lagoa Nova, Natal, Caixa postal 1524 CEP 59078-970, RN, Brazil; eng.renanpires@gmail.com (R.P.A.); maitelli@dca.ufrn.br (A.L.M.); andres@dca.ufrn.br (A.O.S.)

**Keywords:** pipeline inspection, maintenance, electronic board, testing loop, supervisory system

## Abstract

The pipeline inspection using a device called Pipeline Inspection Gauge (PIG) is safe and reliable when the PIG is at low speeds during inspection. We built a Testing Laboratory, containing a testing loop and supervisory system to study speed control techniques for PIGs. The objective of this work is to present and validate the Testing Laboratory, which will allow development of a speed controller for PIGs and solve an existing problem in the oil industry. The experimental methodology used throughout the project is also presented. We installed pressure transducers on pipeline outer walls to detect the PIG’s movement and, with data from supervisory, calculated an average speed of 0.43 m/s. At the same time, the electronic board inside the PIG received data from odometer and calculated an average speed of 0.45 m/s. We found an error of 4.44%, which is experimentally acceptable. The results showed that it is possible to successfully build a Testing Laboratory to detect the PIG’s passage and estimate its speed. The validation of the Testing Laboratory using data from the odometer and its auxiliary electronic was very successful. Lastly, we hope to develop more research in the oil industry area using this Testing Laboratory.

## 1. Introduction

The increasing demand for transport of oil and gas by the petrochemical industry makes it necessary to use oil and gas pipelines. This caused an increase in research and development by petroleum companies, with the objective of improving efficiency, security, reliability and durability of oil and gas pipelines under different operating conditions.

**Figure 1:**
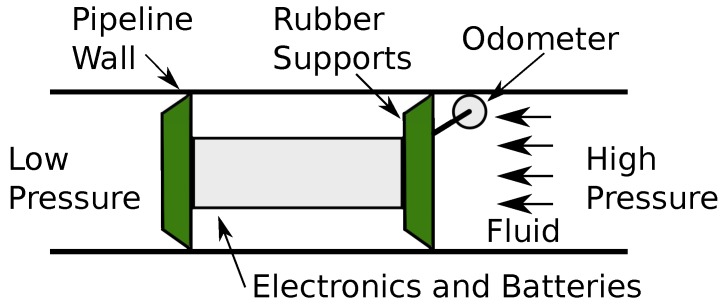
An example of the PIG inserted in the pipeline. Source: Edited by authors.

One way for achieving these improvements is via pipeline inspections and the most used method is pigging. It consists of launching a PIG, see Figure 1, through all pipeline extension to obtain physical integrity information of pipelines. An inspection by PIG is able to collect data about the operating conditions of the pipeline and provide the inspection companies information for decision-making, whether it is preventative or corrective maintenance.

PIGs are pushed by pressure differential (ΔP) produced by fluid flow and can perform: (a) pipeline cleaning and debris removal (Utility PIGs); and (b) data collecting, extension and location of defects within the pipeline (Smart PIGs).

It is common for the PIG to stop during its run because of obstructions within the line, e.g., wax or paraffin. The PIG only resumes its movement when there is enough pressure on its rear. As a result, the speed increases uncontrollably. This event is called “speed excursion” and it is described by [1].

The speed excursion is a speed peak achieved by the PIG, which can cause damage to itself and the pipeline walls [2]. In order to maintain a safe and reliable inspection, the speed of the PIG needs to stay low, and, according to [3], this speed is usually in the range of 1 to 5 m/s inside liquid pipelines and 2 to 7 m/s inside gas pipelines.

Currently, in Laboratorio de Avaliação de Medição em Petróleo (LAMP) in Universidade Federal do Rio Grande do Norte (UFRN), the PIG Speed Control Project is in progress. In the first phase, we built a Testing Laboratory containing a testing loop and a supervisory system. Now, in the second phase, we are validating this Testing Laboratory, comparing data from the supervisory system with data from the prototype PIG, using an odometer to measure its speed. In the third phase, we will test an in-development technology to control the speed of the prototype PIG, containing a bypass valve, electro pneumatic valve and electronic board. In this way, we aim to solve an existing problem in the oil industry, which is to control the speed of the PIGs for safe and reliable inspections.

The uniqueness for this Testing Laboratory is that, in the near future, it will allow the development of a PIG speed estimator using neural networks, to be used in case of a faulty odometer, an accelerometer and gyroscope module to determine the acceleration and the PIG’s trajectory recovery, energy recovery using a small electric generator within the PIG, making use of the internal air flow to rotate a turbine, etc.

The next sections of this article will be organized as follows: in Section 2, we present related work about PIG technology and its current stage. In Section 3, we explain PIG’s movement and working principles of the sensors used in this work. In Section 4, we show the main components for assembly of the Tests Laboratory and PIG prototype. In Section 5, we present the experimental methodology. In Section 6, we show the results from testing loop and prototype PIG. Lastly, in Section 7, the conclusions of this work are discussed.

## 2. Bibliography Review

In this section, the usage of the PIGs in petroleum industry, its speed control and different testing loops will be presented.

### 2.1. Use of PIGs

Various types of PIGs, their importance and the operations they are able to perform in pipeline maintenance are described in [1]. It also presents relevant aspects to be considered in pipeline design in order to allow the passage of PIGs.

Some geometric aspects and the equations for determining the size of the PIG depending on the tubing geometry are addressed by [4].

Besides being widely used tools in the maintenance of the existing pipelines, the PIGs can still be used in the installation of new pipelines and at the end of its lifespan. The role of the tools in the pre-commissioning of new pipelines and the performed operations are described in [5].

The use of sealing PIGs in the process of deactivating pipelines, where it is often necessary to remove the product and fill it with inert gas, is presented in [6].

However, it is worth mentioning that there are other tools of inspection and cleaning of ducts that may be more suitable in certain situations. A robotic tool was developed by Petrobras (Rio de Janeiro, RJ, Brazil), called “GIRINO”, whose movement is based on amphibians, that is able to move in the pipe independently of the direction of the fluid [7].

Several tools for the internal inspection of ducts are described in [8]. The author also presents criteria to assist in choosing the most suitable type for a given operation.

### 2.2. Speed Control of PIGs

Several researchers have addressed the development of speed control techniques for PIGs. According to [9], the PIG velocity in pipelines should not exceed 7 m/s, and he presents a nonlinear control method, besides a mathematical modeling and a simulation of the movement of the tool that includes a speed excursion situation.

In [10], the author presents a history of PIG velocity control, citing US patents in the area and describes a control system that operates through a by-pass valve. In addition, it states that the instrumented PIGs must move at a constant speed to avoid distortions in the collected data, since the sampling time of the acquisition system is constant.

The speed control is important in the various types of PIG according to [11]. Furthermore, they describe several methods of speed control, classifying them as passive or active.

A test bench to simulate a speed excursion situation and the proposal of an on–off control strategy based on Pulse Width Modulation (PWM) is presented by [12].

A linearized model is presented in [13] to estimate the velocity of the PIG and a speed controller based on Fuzzy Logic, whose focus is to minimize the speed excursion.

### 2.3. PIG Testing Systems

A system designed to test various types of PIGs under various flow conditions is addressed by [1]. The system has pressure and flow transducers that are useful in studying the behavior of PIGs. The results indicate that the average speed of the device can be calculated by means of the pressure transducers.

An experimental apparatus with the objective of evaluating models for the removal of paraffin deposits by means of cleaning PIGs, consisting of a ϕ6” steel tube, is presented in [14]. The devices are driven by steel cables attached to a winch, and load cells are used to record the force exerted by the PIGs during the tests.

The work of [15] shows two examples of test pipelines, localized in Norway and Texas (USA).

In [16], the authors present a PIG testing pipeline and its supervisory system. The results demonstrate how the use of pressure sensors can be used to detect the passage of PIG along the pipe as well as its average speed.

## 3. Theoretical Foundation

This section will provide a simple theoretical background for understanding the work, as well as present a simplified model of the PIG’s motion, explain the pressure sensor’s measurement principle and Hall effect sensor working principle.

### 3.1. PIG Movement

**Figure 2:**
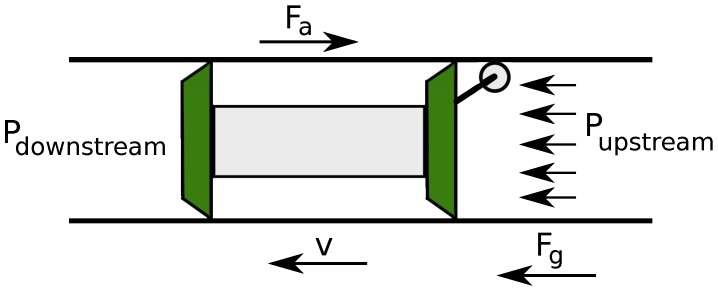
Forces exercised on the PIG. Source: By authors.

Once the PIG is inserted into the pipeline, transported fluid drives the device thanks to pressure difference, as illustrated in Figure 2.

This movement can be explained in terms of Newton’s Second Law of Motion, according to Equations (1)–(3):

(1) $$M\cdot a=F_{g}-F_{a},$$

(2) $$F_{g}=\Delta P\cdot A,$$

(3) $$F_{a}=B\cdot v+F_{s},$$

where *M* is the PIG’s mass; *a* is the PIG’s acceleration; $F_{g}$ is the force that acts in the PIG; $F_{a}$ is the friction between rubber supports and pipeline wall; ΔP is the pressure differential applied on PIG; *A* is the PIG’s back area; *B* is the Viscous friction coefficient; *v* is the PIG’s speed; and $F_{s}$ is the dry friction.

Disregarding the dry friction and replacing $a=\frac{dv}{dt}$, we have:

(4) $$M\cdot \frac{dv}{dt}=\Delta P\cdot A-\left(B\cdot v+0\right),$$

(5) $$M\cdot \frac{dv}{dt}+B\cdot v=\Delta P\cdot A,$$

(6) $$V_{\mathrm{PIG}}=f\left(\Delta P\right).$$

Thus, from Equations (5) and (6), we can state that the PIG’s motion mainly depends on pressure.

### 3.2. Pressure Measurement and Pressure Transducers

The most common types of pressure sensors are the bridge-based or piezoresistive because of their simple construction and durability. Piezoelectric pressure transducers are generally more expensive, but they have superior dynamic response [17].

Piezoelectric pressure sensors, e.g., measure dynamic pressure. They are based on the piezoelectric effect, which is the result of straining a piezo element to generate a voltage.

The pressure transducer we have chosen is the Novus NP-300 (Novus, Porto Alegre, RS, Brazil), suitable for low-pressure applications and with relative low-cost. It has an integrated 2-wire transmitter (4–20 mA).

It uses piezoelectric principles to measure relative pressure inside the pipeline, so it provides a good dynamic response. Its measuring range is 0–1 MPa; the supply voltage is 11–33 Vdc; accuracy <0.25% of upper range limit; dynamic response <30 ms.

### 3.3. Hall Effect Sensor Principle

A Hall effect sensor is a device that detects the magnetic field and varies its output voltage. This variation can be applied to detect the motion of a part of machine. According to [18], it is only necessary to fix a magnet on surface of a spinning component to measure its rotation.

**Figure 3:**
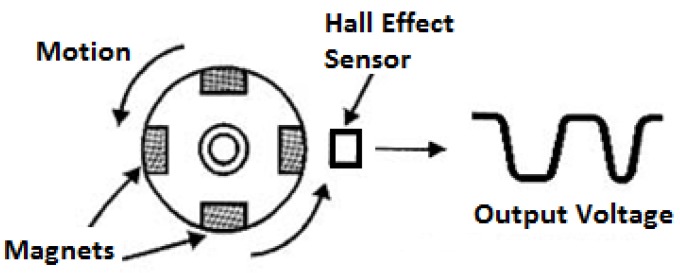
Hall effect sensor under the influence of a magnetic field. Source: Edited by authors.

Figure 3 shows a spinning component and the output voltage of a Hall effect sensor.

This principle is applied to the odometer, where a magnet pass in front of a Hall effect sensor toggles the output voltage. At each complete rotation of the wheel of the odometer, a square pulse is produced, which is read by the microcontroller. This counts the time between pulses and, with the circumference of the wheel of the odometer, it is possible to calculate the speed of the PIG.

The data of speed and travelled distance by the PIG are saved in a memory card and recovered by the PIG complete the race inside the testing loop.

## 4. Proposed System

The system we have constructed consists mainly of a Programmable Logic Controller (PLC), a supervisory system, a prototype PIG and testing loop.

### 4.1. The PLC and Supervisory System

The PLC — in this case, WEG TPW-03 60HT-A — provides the interface between the transducers and the computer. It converts the transducers analog signals to digital signals by means of a 10-bit analog-to-digital converter (ADC). The digital information is then made available to the desktop computer via Modbus protocol.

**Figure 4:**
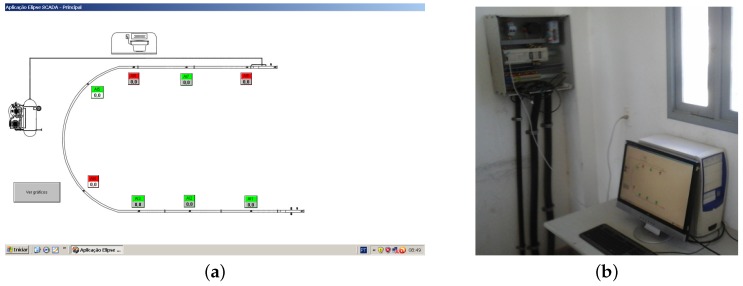
Workstation of supervisory system. (**a**) print screen of supervisory; (**b**) PLC box and desktop computer. Source: By authors.

We have used Elipse SCADA demo version v2.29 (Elipse Software, Porto Alegre, RS, Brazil), running on Microsoft Windows XP™ (Redmond, WA, USA), to retrieve data from the PLC. The application displays time-series of pressures and allows us to store data on the computer hard disk, see Figure 4a.

Figure 4b shows the work station with PLC box and desktop computer.

### 4.2. Prototype PIG

A prototype PIG was acquired by LAMP for execution of experimental tests of speed control, using the testing loop. The device is comprised of two polyurethane piston cups of ϕ6” installed at the PIG’s edge, an electronic capsule of steel installed in the middle of the PIG, and an odometer installed in the rear part of it.

**Figure 5:**
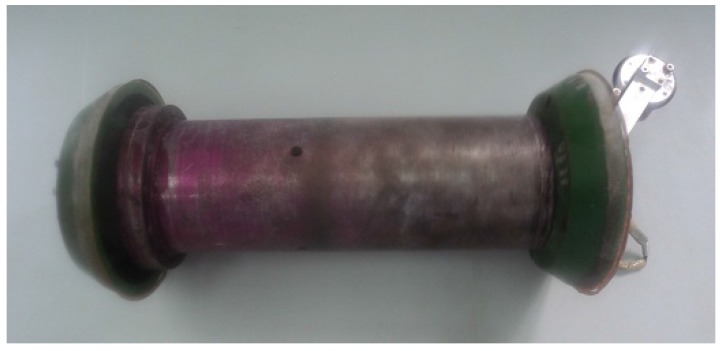
Prototype PIG. Source: By authors.

Figure 5 presents the prototype PIG used in this work for the experimental tests.

### 4.3. The Testing Loop

A testing loop was constructed with steel pipelines of ϕ6” and ϕ8”, thickness of 5 mm and total extension of 55 m, at the premises of LAMP/UFRN. It is filled with compressed air up to 6 bar. This testing loop was mounted with the objective of running the prototype PIG and developing its speed controller. However, we are still on the testing loop validation phase, and only then will we proceed with other research.

**Figure 6:**
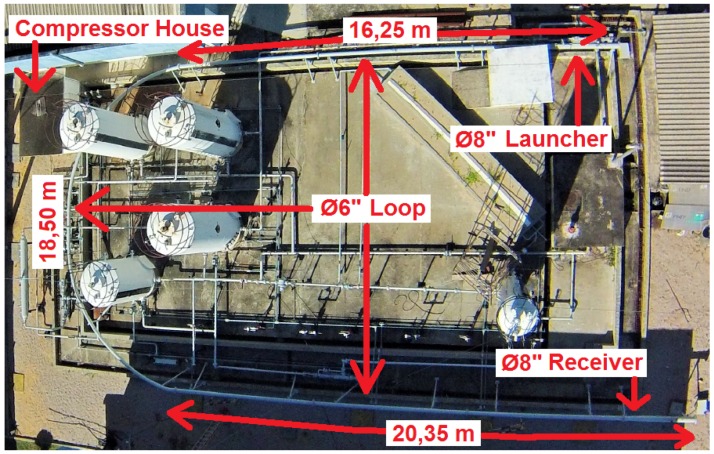
Testing loop in the aerial view. Source: Edited by authors.

Figure 6 shows the aerial view of the testing loop, with a highlight on the ϕ8” launcher, on the “U” shape ϕ6” testing loop, compressor house and ϕ8” receiver.

The operation principle of testing loop is based on a pressure increase after the PIG goes past the pressure transducers.

We installed seven pressure transducers in the testing loop in order to increase system reliability, improve the monitoring of the PIG’s movement within the testing loop and gather better data from the pressures’ transducers. In case of faults in one or two transducers, we would still have enough data to analyze.

**Figure 7:**
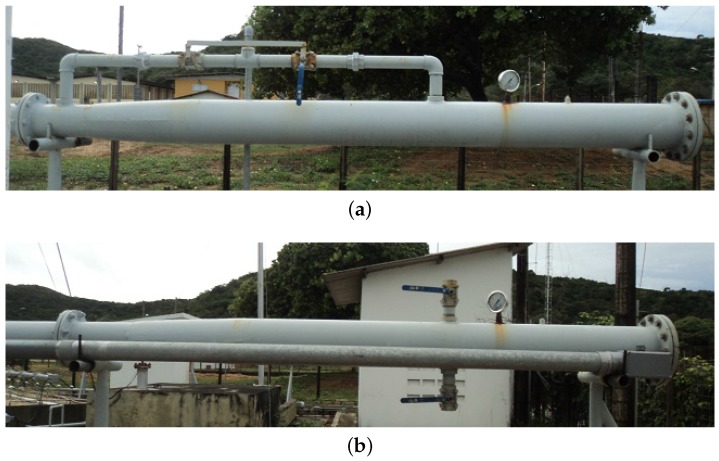
Lateral view. (**a**) lateral view of the ϕ8” launcher; (**b**) lateral view of the ϕ8” receiver. Source: By authors.

The ϕ8” launcher (Figure 7a) is used to insert the prototype PIG into the tests loop and has one manometer and two ball valves. ϕ8” receiver (Figure 7b) is used to remove the prototype PIG from the testing loop and has two ball valves.

**Figure 8:**
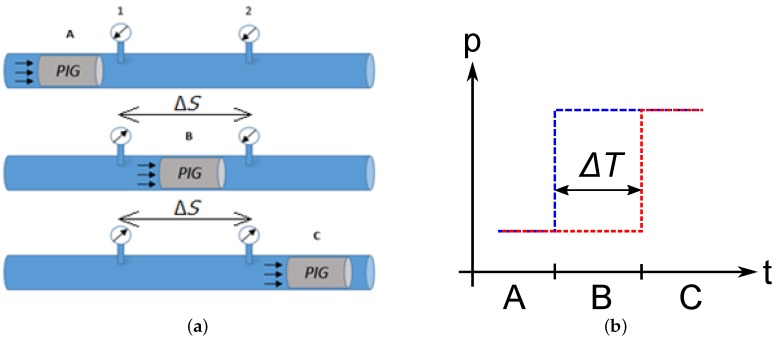
Response and behavior of transducers. (**a**) pipeline stretch with two transducers; (**b**) pressure response. Source: Adapted from [15].

Figure 8a shows the expected behaviour of pressure in the pipeline based in two transducers and their pressure response versus time (see Figure 8b). At first, the measurement pressure by two transducers would be equal, so the curves would be overlapping (Region A). Later, when the PIG passes by the first transducer, pressure increases (Region B). Lastly, when the PIG passes by the second transducer, the pressure measurement of both transducers should be the same (Region C).

Therefore, knowing the time range between two pressure measurements (ΔT) and the distance between two sensors (ΔS), it is possible to calculate average speed for the PIG through the equation $V_{\mathrm{PIG}}=\frac{\Delta S}{\Delta T}$.

## 5. Methodology

In this section, we will present the methods applied for assembling the prototype PIG and proceedings to use the testing loop.

### 5.1. Installation of the Electronic Board and Odometer in the PIG

To install the electronic board and batteries into the PIG’s body, the back cover of PIG was removed. The components were installed and the back cover returned to its place. Then, the odometer was installed on the back cover of the PIG.

Then, the PIG is taken to the ϕ8” launhcer to perform the experimental tests.

### 5.2. PIG Running in the Testing Loop

**Figure 9:**
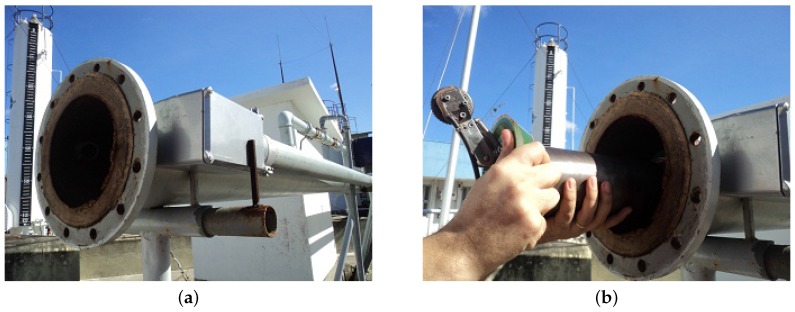
Procedures with the prototype PIG in the ϕ8” launcher. (**a**) opening the ϕ8” launcher cover; (**b**) inserting the prototype PIG in the ϕ8” launcher. Source: By authors.

**Figure 10:**
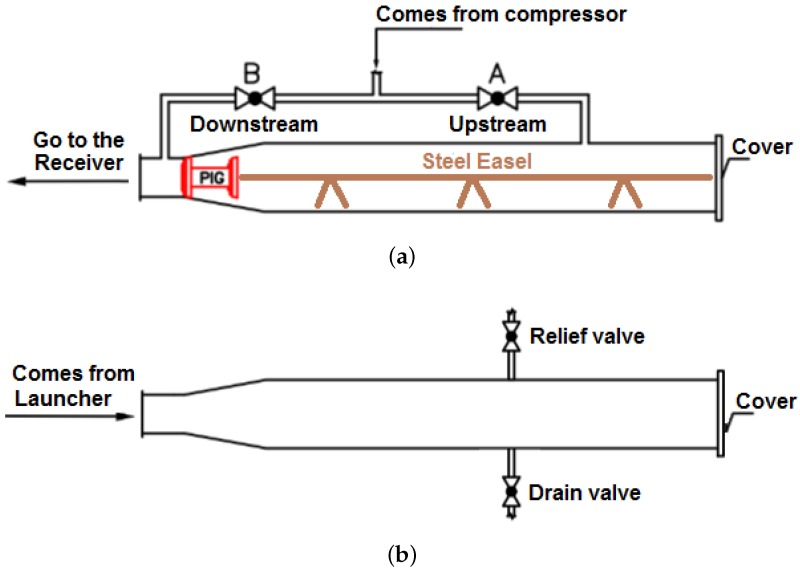
Continuation of procedures with PIG. (**a**) opening ball valves in the ϕ8” launcher; (**b**) opening ball valves in the ϕ8” receiver. Source: Adapted From [15].

To perform the prototype PIG run using the testing loop, it was necessary to open the ϕ8” launcher cover (Figure 9a). The prototype PIG was then input into the ϕ8” launhcer (Figure 9b) and together with a steel easel (Figure 10a). The steel easel is the accessory that prevents a backward movement of the PIG. Lastly, the ϕ8” launcher was closed.

Within the ϕ8” launcher, the ball valve B was opened and ball valve A was closed (Figure 10a). The compressor was turned on and the downstream pipeline was pressurized with compressed air with 5.5 bar. The ball valve A was opened and ball valve B was closed (Figure 10a). The upstream pipeline was pressurized with 5.5 bar of compressed air.

While the pipeline was being pressurized, the pressure was monitored through the supervisory, which received the transducers’ signals and displayed the results on the computer screen.

With the downstream and upstream pressures equalized, the drain valve (Figure 10b) present in the receiver was opened, resulting in a pressure differential that moved the PIG forward.

The speed was acquired by the microcontroller through the odometer’s signals.

**Figure 11:**
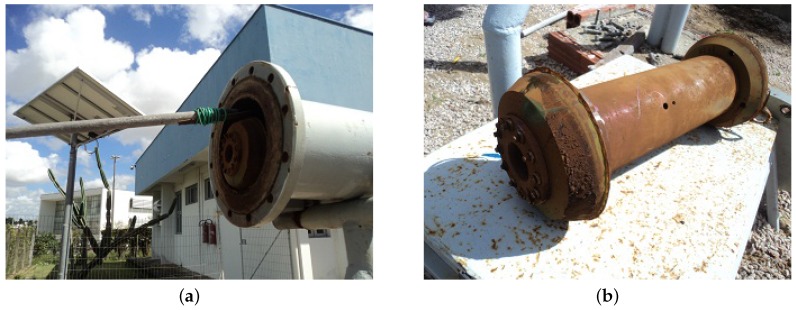
Procedures with the prototype PIG in the ϕ8” receiver. (**a**) inserting a hook to pull the PIG; (**b**) PIG out from the receiver. Source: By authors.

After the prototype PIG reached the end of the testing toop, the receiver cover was opened and a hook was used to pull out (Figure 11a). A rust layer was verified once the PIG was checked (Figure 11b). In possession of the PIG, its back was opened to allow access to the data in the memory card, and then it was cleaned with a cloth and closed again.

## 6. Results and Discussion

**Figure 12:**
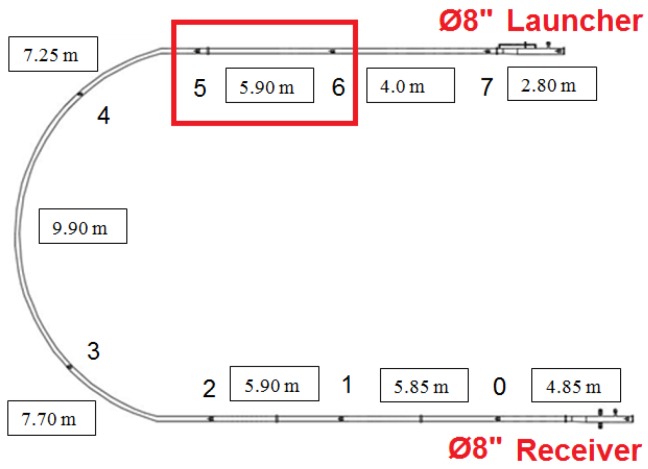
Position of the transducers analyzed in this work. Source: By authors.

Figure 12 illustrates the position of the transducers installed in the testing loop. The section of the test line analyzed in this work is marked, which is comprehended between the transducers 6 and 5.

**Figure 13:**
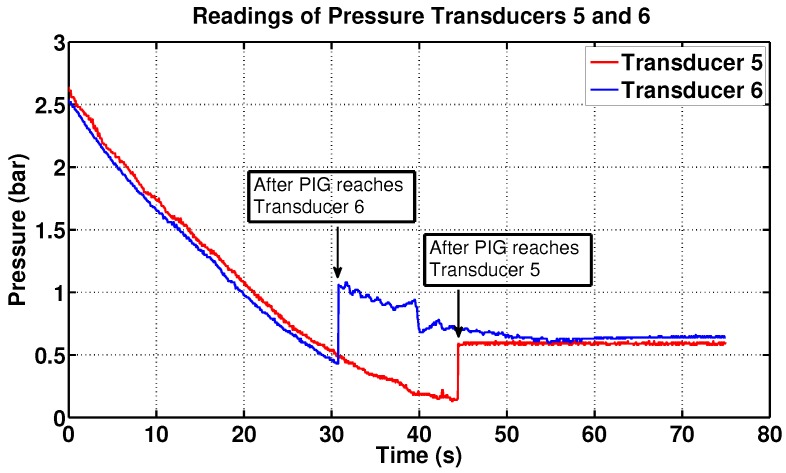
Supervisory data of PIG’s passage between the transducers 6 and 5. Source: By authors.

In addition, 272 pressure readings were taken by the supervisory system while the PIG passed from the 6th to the 5th transducer (Figure 13). Since each pressure reading was taken every 50 ms, it was verified that the PIG went through the distance between the sensors (5.90 m) in 13.6 s. Thus, the average speed of the PIG in this section was 0.43 m/s.

**Figure 14:**
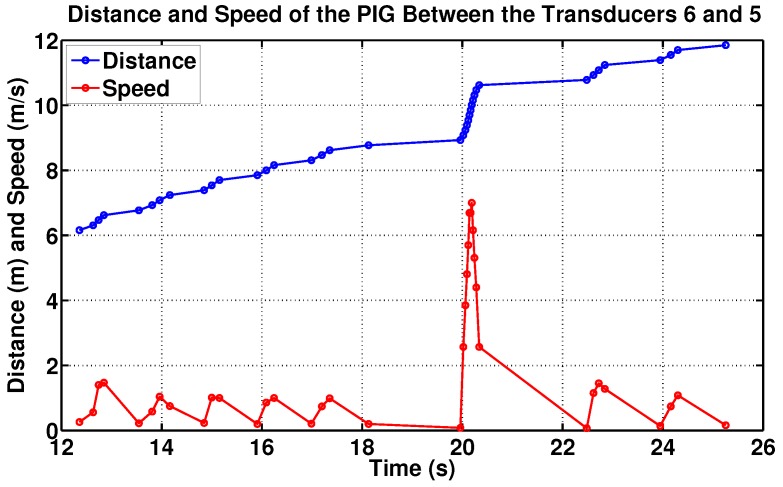
Speed PIG between the transducers 6 and 5, according the odometer. Source: By authors.

Based on the memory card data, it was verified that the PIG travelled 5.70 m in 12.53 s, resulting in an average speed of 0.45 m/s (Figure 14). The speed value delivered by the odometer is more reliable because it is a direct reading, while the value delivered by supervisory is indirect, a estimative.

Comparing the speed results from the supervisory (0.43 m/s) and memory card (0.45 m/s), assuming this value as the most faithful, it was verified that the error between the values was 4.44%, which is experimentally acceptable.

Lastly, the results from others transducers were not shown in order to make the graph clearer.

## 7. Conclusions

Through the results presented in this work, the practical utility of the Testing Laboratory built for the research and better comprehension of PIG movement inside a pipeline was observed. Speed data was gathered by the prototype PIG and stored in its memory. It was then verified by the estimative from the supervisory, which calculated the average speed through the transducer’s pressure readings. The error of the values obtained was relatively small and validated the Testing Laboratory with success. The main contribution this Testing Laboratory will be in the estimation of average speed of utility PIGs (e.g., foam PIGs, solid cast PIGs, spherical PIGs, and others), which do not have an odometer or an electronic board. Lastly, as future work, the following could be researched: a PIG speed controller, a speed estimator through neural networks, trajectory mapping, and others.
